# On the Alteration of the Properties of Sulphate of Quinine by Its Mixture with Coffee

**Published:** 1848-05

**Authors:** 


*On the alteration of the Properties of Sulphate of Quinine by its mixture with Coffee.* By M. C. F.—It has been before advanced, that sulphate of quinine, mixed with an aqueous infusion of coffee, undergoes a certain decomposition; one part of the salt forming with the tannin of the coffee an insoluble combination; another part remaining mingled in the liquid, united to a fatty oil and vegetable extractive; the third being dissolved by the free acids formed in the liquid. M. C. F. has positively proved by clinical experiments, that these different reactions in reality diminish the ordinary curative powers of the medicine. Thus, he treated a regular tertian fever, for which he ordered twenty-eight grains of sulphate of quinine in an infusion of coffee; the effects of the remedy did not tally with what is generally observed, in consequence of a like dose in similar circumstances, for the febrile paroxysm was reproduced with the same regularity. A second dose of the salt was then administered, but dissolved by sulphuric acid; the fever was immediately cut short.

In another case, he made twenty grains of sulphate of quinine be taken in a cup of coffee by a patient affected with intermittent suborbital neuralgia, but the painful paroxysm returned the same as if no anti-periodic had been given. The second observation will, without doubt, be remarked as proving less than the first, the counter-proof which made all the force of the first having failed in this ; how often, in fact, have we not seen quinine fail even in those where the intermittent character was more regular, and with the best mode of administering the remedy.—*Ibid.*

